# Severe Personality Disorder with Chronic Suicidal Ideation Treated through an Intensive Acute Care Program

**DOI:** 10.1192/j.eurpsy.2025.515

**Published:** 2025-08-26

**Authors:** T. Bollain Muñoz, I. Oliveira Amat, M. Valtueña Garcia, M. L. Barrigón Estevez, J. Sanchez Adsuara, R. Crespo Rubio

**Affiliations:** 1 Psychiatry; 2 HGUGM, Madrid, Spain

## Abstract

**Introduction:**

34-year-old patient with multiple sporadic and brief contacts with mental health services, which he unilaterally chooses to discontinue. He has a long history of parasuicidal behavior dating back to adolescence. The patient does not report any prior diagnoses and has no history of inpatient admissions. The patient describes experiencing social isolation, lacking contact with his family of origin, and having no significant peer relationships.

**Objectives:**

The primary goal is to improve the patient’s engagement with mental health services, particularly in a case experiencing chronic, unaddressed symptoms, by utilizing intensive and structured programs. An additional objective is to address the patient’s self-identification with suicidal ideation.

**Methods:**

The patient’s first contact with mental health services in this region of Spain was through the emergency department following a suicidal episode. During this encounter, the clinician introduced an intensive program designed to address suicidal ideation through regular visits over a set period. The patient agreed to participate and was subsequently enrolled in the PRISURE program at HGUGM in Madrid, where he received multiple sessions each month (between 2 and 4) with both a psychiatrist and a nurse from March to June 2024.

**Results:**

At the beginning of the program, the patient was fixated on the idea of suicide from a romantic/nihilistic perspective, displaying a pervasive rejection of interpersonal contact and a narcissistic element in interactions. He expressed persistent suicidal ideation. Over the course of frequent visits, the patient gradually began to connect with the chronic nature of his behaviors and started to identify additional symptoms. Despite partial engagement in the program, in this case with some missing consultations, his attendance at consultations improved significantly compared to his prior behaviors. An inpatient stay was initially offered and declined by the patient; however, after further consideration, he later presented to the emergency room and agreed to inpatient treatment. During the admission, a diagnostic assessment was carried out and discussed with the patient, revealing challenges in identity, object relations, and moral functioning, which were positioned within the spectrum of personality disorders, particularly highlighting narcissistic and antisocial traits.

**Conclusions:**

Initially focused on his suicidal ideation, the patient, through the PRISURE program, gradually explored underlying difficulties contributing to his suicidal behavior. This process allowed him to accept further support, ultimately leading to an inpatient stay. Potential diagnoses were discussed openly with the patient, helping him to gain a clearer understanding of his lifelong challenges and enabling him to articulate these difficulties within the therapeutic context.

**Disclosure of Interest:**

None Declared

